# Supplementary material for: Morphopathogenesis of Adult Acquired Cholesteatoma
Source: Medicina (Kaunas). 2023 Feb 7;59(2):306. doi: 10.3390/medicina59020306 (PMC9960810; doi:10.3390/medicina59020306)
Supplement: Supplementary file 1 [file medicina-59-00306-s001.zip › medicina-2162931-supplementary.pdf]

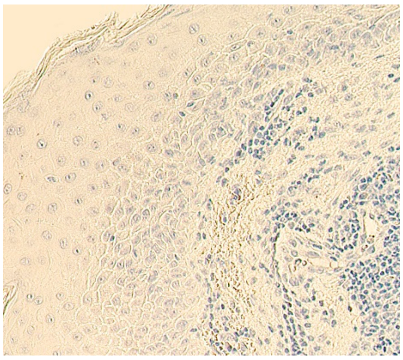

Figure S1. The example of test sample of the cholesteatoma: MMP2 negative control in cholesteatoma tissue. X 250. Positive controls in accordance to the companies guidelines and negative controls with excluding of primary antibody were developed.

Figure S2. Pictures of each criterion of semi-quantitative method.

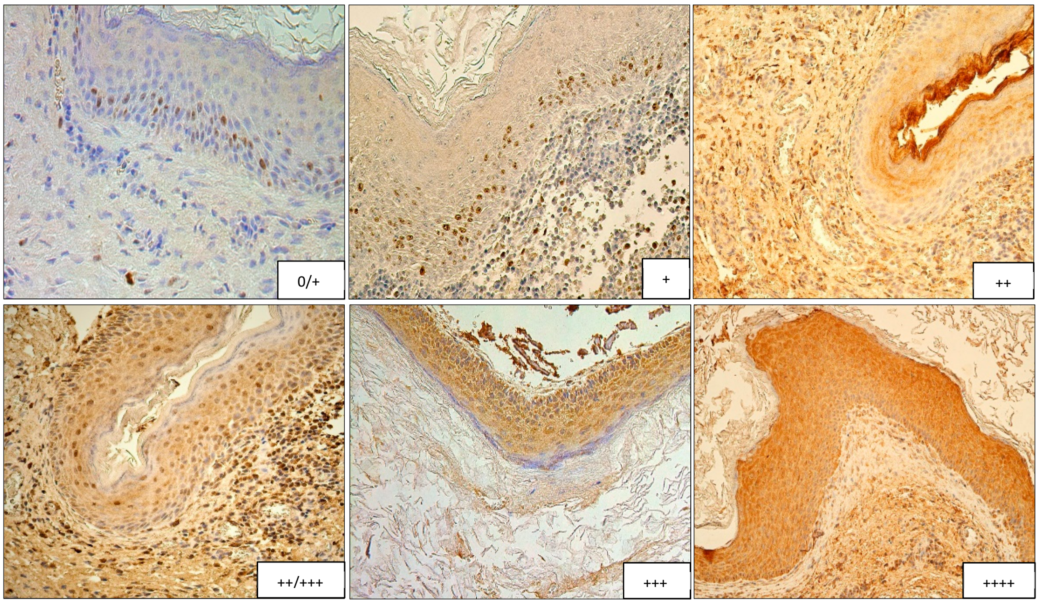

0/+ = occasional positive structures; **+** = few positive structures; **++** = moderate number of positive structures; **++/+++** = moderate to numerous positive structures; **+++** = numerous positive structures; **++++** = an abundance of positive structures in the visual field.

Figure S2. Pictures of each criterion of semi-quantitative method.

Table S1. Additional statistical information.

Group			MMP-2 M	MMP-2 P	MMP-9M	MMP-9P	TIMP-2 M	TIMP-2 P	TIMP-4M	TIMP-4P	Shh M	Shh P	IL-1 M	IL-1 P	IL-10 M	IL-10 P	NFKB M	NFKB P	Ki-67 M	Ki-67 P	VEGF M	VEGF P	HBD-2 M	HBD-2 P	HBD-4 M	HBD-4 P
**Patients**	N	Valid	19	19	19	19	19	19	19	19	19	19	19	19	19	19	19	19	19	19	19	19	19	19	19	19
		Missing	0	0	0	0	0	0	0	0	0	0	0	0	0	0	0	0	0	0	0	0	0	0	0	0
	Mean		1.5437	1.3332	0.4211	0.7189	0.8505	0.5953	2.5000	2.0526	2.4737	1.8158	1.2632	1.1137	1.4737	1.2453	1.9737	1.2716	0.6568	0.5605	1.6837	0.6926	1.5526	1.0874	0.7805	0.5863
	Std. Deviation		0.86064	0.61644	0.53394	0.43442	0.94937	0.60230	0.81650	0.72447	0.71635	0.58239	0.91846	0.76020	1.03379	0.82512	0.63407	0.96455	0.32692	0.47580	0.89646	0.68121	0.59849	0.63477	0.95113	0.50496
	Minimum		0.00	0.33	0.00	0.00	0.00	0.00	0.00	0.00	1.00	0.50	0.00	0.00	0.00	0.00	1.00	0.00	0.33	0.00	0.33	0.00	0.50	0.00	0.00	0.00
	Maximum		3.50	3.00	2.00	1.50	3.00	2.00	3.50	3.00	4.00	3.00	3.00	3.00	3.00	3.00	3.00	3.00	1.50	1.50	3.00	2.00	2.50	2.00	3.00	2.00
**Control**	N	Valid	7	7	7	7	7	7	7	7	7	7	7	7	7	7	7	7	7	7	7	7	7	7	7	7
		Missing	0	0	0	0	0	0	0	0	0	0	0	0	0	0	0	0	0	0	0	0	0	0	0	0
	Mean		1.0714	0.9286	1.0714	0.6900	1.5714	0.7143	2.1429	1.6429	1.6429	0.6429	0.6429	0.7143	1.7857	1.5000	0.7143	0.4757	0.0943	0.0943	2.4286	0.7857	1.0471	0.2614	1.2143	0.5714
	Std. Deviation		1.20515	0.18898	0.44987	0.41356	0.78680	0.39340	0.69007	0.47559	1.43510	0.47559	0.55635	0.26726	0.63621	0.64550	0.69864	0.50411	0.16102	0.16102	0.44987	0.48795	0.69921	0.25182	0.39340	0.34503
	Minimum		0.00	0.50	0.50	0.33	0.50	0.00	1.00	1.00	0.00	0.00	0.00	0.50	1.00	0.50	0.00	0.00	0.00	0.00	2.00	0.00	0.33	0.00	1.00	0.00
	Maximum		3.00	1.00	1.50	1.50	2.50	1.00	3.00	2.00	3.50	1.00	1.50	1.00	2.50	2.00	2.00	1.50	0.33	0.33	3.00	1.50	2.00	0.50	2.00	1.00
